# Supplementary material for: A general framework to support cost-efficient survey design choices for the control of soil-transmitted helminths when deploying Kato-Katz thick smear
Source: PLoS Negl Trop Dis. 2023 Jun 22;17(6):e0011160. doi: 10.1371/journal.pntd.0011160 (PMC10321644; doi:10.1371/journal.pntd.0011160)
Supplement: S1 Info — (DOCX) [file pntd.0011160.s001.docx]

**A general framework to support cost-efficient survey design choices for the control of soil-transmitted helminths when deploying Kato-Katz thick smear**

**S1 Info: Quantification of between and within -school variation in faecal egg counts**

Adama Kazienga^1,2*^, Bruno Levecke^1^, Gemechu Tadesse Leta^3^, Sake J. de Vlas^2^ , Luc E. Coffeng^2^

^1^Department of Translational Physiology, Infectiology and Public Health, Ghent University, Merelbeke, Belgium

^2^Department of Public Health, Erasmus MC, University Medical Center Rotterdam,

Rotterdam, The Netherlands

^3^Bacterial, Parasitic and Zoonotic Diseases Research Directorate, Ethiopian Public Health Institute, Addis Ababa, Ethiopia

### *Corresponding author: [kazienga_adama@yahoo.fr](mailto:kazienga_adama@yahoo.fr).

This supplement describes how we quantified variation in faecal egg counts (FEC) for soil-transmitted helminths (STH) between and within schools, based on Ethiopian pre-control FEC data from school-aged children [1]. This dataset included egg count data from 150,256 individuals from 2,921 schools from 619 woredas (administrative units), based on single Kato-Katz (KK). For our analyses we only used the subset of data from woredas in which all schools included at least one egg-positive individual (**Table A in S1 Info** ). This yielded very similar estimates of variation within and between schools as when analysing data from all woredas with at least one egg-positive individual (which could include schools in which all children tested negative), but was computationally much faster, facilitating the exploration of a wider range of modelling assumptions.

**Table A. Number of datapoints used per soil-transmitted helminth species.** These numbers constitute a selection of the subset of data from woredas in which all school included at least one egg-positive individual, based on single Kato-Katz.

| **Worm species** | **Number of individuals** | **Number of schools** | **Number of woredas** |
| --- | --- | --- | --- |
| Hookworm | 41,797 | 776 | 162 |
| *Ascaris lumbricoides* | 70,488 | 1,324 | 277 |
| *Trichuris trichiura* | 35,090 | 659 | 142 |

In **Figure A in S1 Info** , we provide an overview of the selection of data used for the analysis, summarised in terms of the average infection level per woreda (horizontal axis) and the variation between schools per woreda (vertical axis), by STH species. We observe that the between-school variation in mean FEC (standard deviation on the logarithmic scale) is very similar across the range of mean woreda-level egg counts in the data. If anything, for *Ascaris lumbricoides* there might be a slight decline in the level of between-school variation as the woreda-level mean infection level increases.


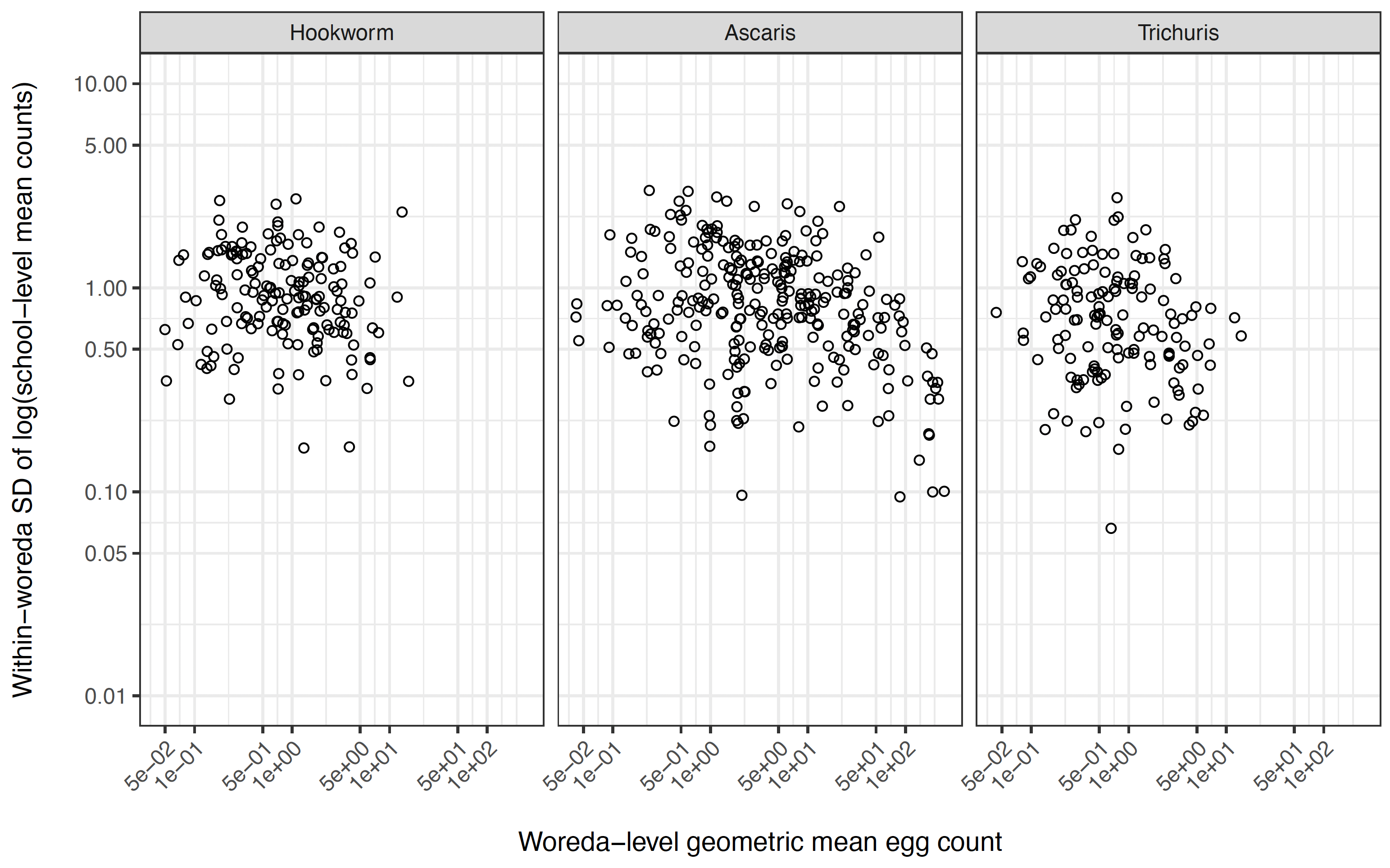


**Figure A. Woreda-level summary of faecal egg count data in terms of geometric mean and standard deviation on the logarithmic scale.** The plotted summaries are based on a selection of the subset of data from woredas in which all school included at least one egg-positive individual, based on single Kato-Katz. Note that both axes are logarithmic.

The summary of the data in **Figure A in S1 Info**  motivated the following specification of a model for quantification of variation between and within schools.

| ${FEC}_{ijk} \sim\mathrm{NB}\left( \mu_{jk},k_{jk} \right)$ | Eq. S1.1 |
| --- | --- |
| $\mu_{jk} \sim\mathrm{lognormal}\left( \log\left( \mu_{k} \right),\sigma_{k} \right)$ | Eq. S1.2 |
| $\mu_{k} \sim\mathrm{lognormal}\left( \log\left( M \right),\Sigma\right)$ | Eq. S1.3 |

Here, the ${FEC}_{ijk}$ of individual $i$ in school $j$ in woreda $k$ is assumed to follow a negative binomial distribution with school-level arithmetic mean $\mu_{jk}$ and shape parameter $k_{jk}$ (Eq. S1.1). The school-level arithmetic means $\mu_{jk}$ in woreda $k$ are assumed to follow a lognormal distribution with woreda-level geometric mean $\mu_{k}$ and standard devation $\sigma_{k}$ (Eq. S1.2). In turn, the woreda-level geometric mean $\mu_{k}$ is assumed to follow a lognormal distribution with overall geometric mean $M$ and standard deviation $\Sigma$ (Eq. S1.3). For the distribution of school-level mean FEC in a woreda (Eq. S1.2), we also considered a gamma distribution, which performed similarly to the log-normal distribution, but was computationally much more expensive, which prohibited the exploration of different model versions. Therefore, from here on, we focus only on the lognormal distribution for school-level mean FEC.

Exploratory analyses showed that overdispersion of mean school-level FECs ($\sigma_{k}$) could safely be assumed to be fixed. Explicitly modelling the gentle slope in between-woreda variation in the *Ascaris lumbricoides* data (**Figure A in S1 Info** ) led to a paradoxical spurious association in which the between-woreda variation actual increased with woreda-level mean FECs (with slope non-significantly different from zero).

The overdispersions of FECs between individuals in the same school ($k_{jk}$) was assumed to be either the same for all observations (“fixed”), or was allowed to vary with school-level infection levels, as has been previously observed in the TUMIKIA study in Kenya [2]. In our model, for this association we defined school-level overdispersion ($k_{jk}$) as a linear function of school-level mean FECs ($\mu_{jk}$):

| $k_{jk}=\beta_{k0}+\beta_{k1}\cdot\mu_{jk}$ | Eq. S1.4 |
| --- | --- |

The slope parameters ($\beta_{\sigma1}$, $\beta_{k1}$) of Eq. S1.4 were restricted to be strictly positive, such that the level of overdispersion would decline with increasing mean infection level. We also considered and explored an alternative specification in the form of
$\log\left( k_{jk} \right)=\beta_{k0}+\beta_{k1}\cdot\log\left( \mu_{jk} \right)$, but this was outperformed by the linear function in terms of predictive performance.

The statistical models were fitted to the data in Stan, using the R package *rstan*. Model performance was assessed by means of mixed posterior predictive checks (mPPC), which constituted repeatedly simulating school-level means and individual-level egg counts from the fitted model (conditional on posterior estimates of woreda-level geometric means and overdispersion) and then comparing those simulated data to the originally observed data (e.g., the distribution of woreda-level mean FECs).

## **Fixed overdispersion within schools**

When assuming that overdispersion of FEC within schools (i.e., between individuals in the same school; $k_{jk}$) are the same for all observations, mPPC of the woreda and school-level means and prevalences showed that the model could reproduce mean FECs but was not able to accurately reproduce the distribution of prevalences in the data. For example, for *Ascaris lumbricoides*, the cumulative distribution of predicted school-level (and thus woreda-level) prevalences of egg-positivity strongly deviated from the observed data (**Figure B in S1 Info** ).


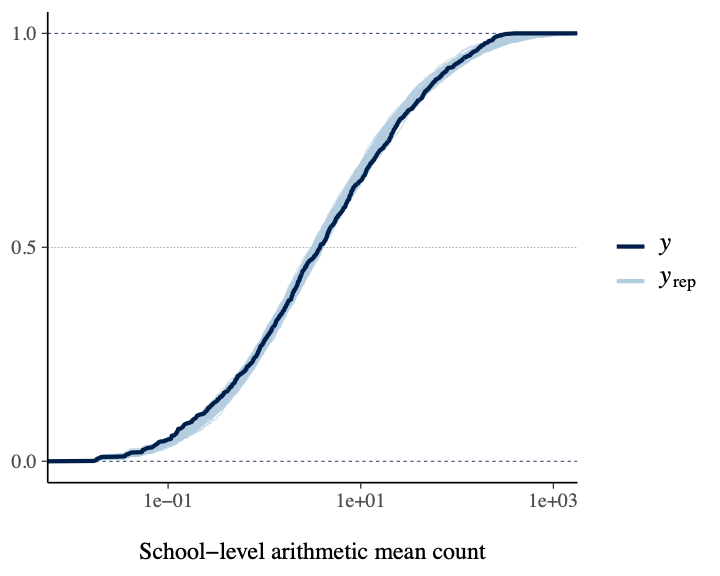

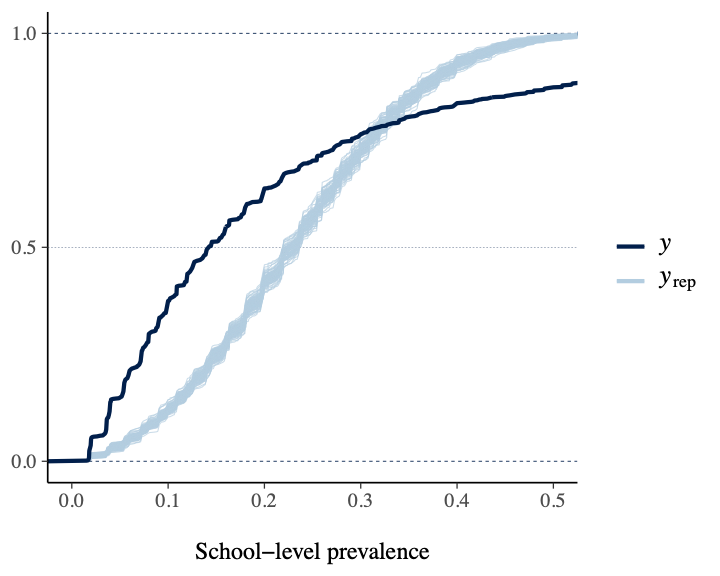


**Figure B. Mixed posterior predictive checks for the assumption of fixed overdisperions of faecal egg counts between and within schools for Ascaris lumbricoides.** The dark blue solid lines represent the cumulative distribution of the observed data ($y$); light blue lines represent repeated simulated datasets ($y_{rep}$), generated from the posterior distribution of parameter values, resampling school-level mean FECs and individuals FECs.

## **Varying overdispersion within schools and fixed overdispersion between schools**

When assuming that overdispersion of FEC is fixed between schools ($\sigma_{k}$) but varies within schools ($k_{jk}$) as a linear function of the school-level arithmetic mean FEC ($\mu_{jk}$), the model adequately reproduced the distribution of mean FECs and prevalences in the observed data (**Figure C in S1 Info**). **Figure D in S1 Info**  below summarises the model-estimated woreda and school-level overdispersion in terms of posterior mean (i.e., the values that were used in the simulation study describes in the main paper), and illustrates the agreement between estimated and observed woreda-level variation in school-level arithmetic mean FECs.


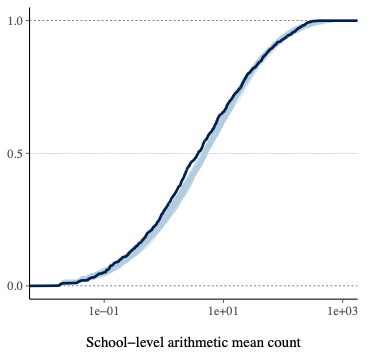

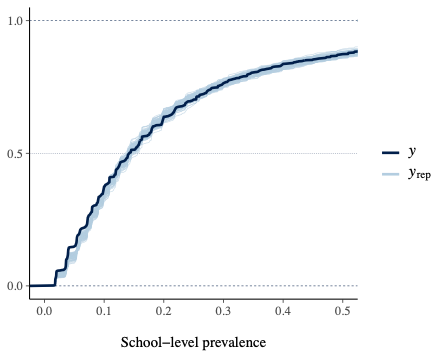


**Figure C. Mixed posterior predictive checks for the assumption of fixed overdisperions of faecal egg counts between schools and varying overdisperions within schools for Ascaris lumbricoides.** The dark blue solid lines represent the cumulative distribution of the observed data ($y$); light blue lines represent repeated simulated datasets ($y_{rep}$), generated from the posterior distribution of parameter values, resampling school-level mean FECs and individuals FECs.


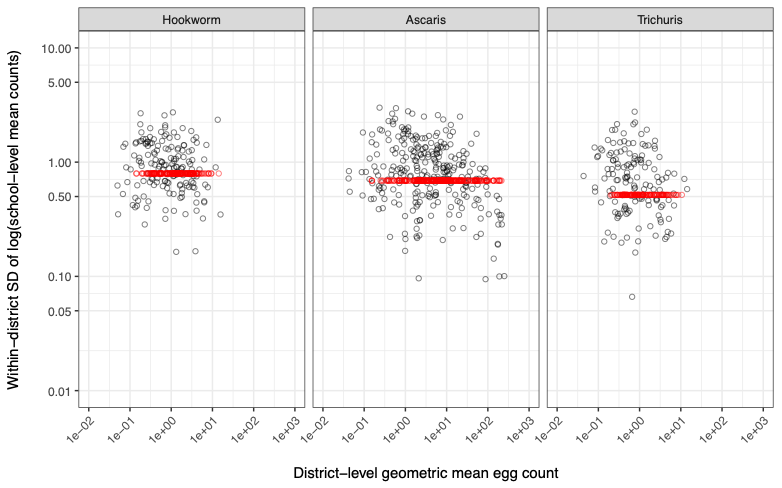

$$\boldsymbol{\sigma}_{\boldsymbol{k}}\boldsymbol{=0.80}$$

$$\beta_{k0}=0.01624$$

$$\beta_{k1}=0.02228$$

$$\boldsymbol{\sigma}_{\boldsymbol{k}}\boldsymbol{=0.69}$$

$$\beta_{k0}=0.01589$$

$$\beta_{k1}=0.00193$$

$$\boldsymbol{\sigma}_{\boldsymbol{k}}\boldsymbol{=0.52}$$

$$\beta_{k0}=0.00983$$

$$\beta_{k1}=0.04444$$

**Figure D. Predicted (red circles) versus observed (black) woreda-level geometric mean and standard deviation of mean school-level faecal egg counts.** Each panel also includes the estimated posterior mean values for the woreda-level overdisperion ($\sigma_{k}$, assumed to be fixed across woredas) and the intercept ($\beta_{k0}$) and slope ($\beta_{k1}$) for the linear association between school-level arithmetic mean ($\mu_{jk}$, as predictor) and overdispersion ($k_{jk}$) of faecal egg counts.

**References**

1. Leta GT, Mekete K, Wuletaw Y, Gebretsadik A, Sime H, Mekasha S, et al. National mapping of soil-transmitted helminth and schistosome infections in Ethiopia. Parasit Vectors. 2020;13: 1–13.

2. Truscott JE, Ower AK, Werkman M, Halliday K, Oswald WE, Gichuki PM, et al. Heterogeneity in transmission parameters of hookworm infection within the baseline data from the TUMIKIA study in Kenya. Parasit Vectors. 2019;12: 1–13.
