# Supplementary material for: A general framework to support cost-efficient survey design choices for the control of soil-transmitted helminths when deploying Kato-Katz thick smear
Source: PLoS Negl Trop Dis. 2023 Jun 22;17(6):e0011160. doi: 10.1371/journal.pntd.0011160 (PMC10321644; doi:10.1371/journal.pntd.0011160)
Supplement: S2 Info — (DOCX) [file pntd.0011160.s002.docx]

**A general framework to support cost-efficient survey design choices for the control of soil-transmitted helminths when deploying Kato-Katz thick smear**

**S2 Info: The mathematical backbone of the simulation framework**

Adama Kazienga^1,2*^, Bruno Levecke^1^, Gemechu Tadesse Leta^3^, Sake J. de Vlas^2^ , Luc E. Coffeng^2^

^1^Department of Translational Physiology, Infectiology and Public Health, Ghent University, Merelbeke, Belgium

^2^Department of Public Health, Erasmus MC, University Medical Center Rotterdam,

Rotterdam, The Netherlands

^3^Bacterial, Parasitic and Zoonotic Diseases Research Directorate, Ethiopian Public Health Institute, Addis Ababa, Ethiopia

### *Corresponding author: [kazienga_adama@yahoo.fr](mailto:kazienga_adama@yahoo.fr).

Our framework simulates egg count from compound lognormal-gamma-gamma-gamma-Poisson distribution. Additionally, the framework captures various sources of variability in egg count across soil-transmitted helminth (STH) species (across schools, between and within individuals, and between repeated smears). We defined the mathematical backbone of the framework as the simulated baseline egg counts in school $j$, individual $k$, day $d$, and sample $s$ for a particular STH species as follows:

(**Eq 1**) $\mu_{j} \sim logN (\mu, \sigma^{2})$

$\mu_{jk} \sim\Gamma\left( k_{k},\frac{\mu_{j}}{k_{k}} \right)$

$\mu_{jkd} \sim\Gamma\left( k_{d},\frac{\mu_{jk}}{k_{d}} \right)$

$\mu_{jkds} \sim\Gamma(k_{s},\frac{\mu_{jkd}}{k_{s}}*w_{sample})$

$X_{jkds} \sim Pois (\mu_{jkds})$

where, $\mu_{j}$ represents the average baseline fecal egg counts (FECs) in egg per gram stool (EPG) for school $j$,$\mu_{jk}$is the expected FEC for an individual $k$ in school $j$, $\mu_{jkd}$represents the expected FEC in individual $k$ for a particular day $d$, and $\mu_{jkds}$ is the expected egg count in an aliquot of stool when deploying Kato-Katz thick smear method (KK). Additionally, $logN$, $\Gamma$ and $Pois$ indicate the lognormal, gamma and Poisson distributions, respectively. The lognormal distribution was parameterized using the mean and the standard deviation on the logarithmic scale. The gamma distribution was parameterized in terms of the shape parameter $k$ and the scale ($\frac{\mu}{k}$), where $\mu$ is the distribution's mean. To quantify the shape parameter of each gamma distribution, the coefficient of variation ($cv$, where shape $k=cv^{-2}$) was used as standardized measure of variability, after Denwood et al. [1]. We estimated the species-specific $k$ values (inter-individual variability $k_{k}$, day-to-day variability $k_{d}$ within individuals, and variability $k_{s}$ between repeated aliquots from the same stool sample) using data from previously published studies [2,3]. The between-school variation $\sigma$ was quantified using the national mapping of soil-transmitted helminths and schistosome infections in the Ethiopia dataset [3] (for details, see **S1 Info**). Additionally, we determined the relationship between the school-level mean EPG and the shape parameter $k_{k}$ (see **S1 Info** for more details**)**, which was defined as follows:

(**Eq 2**) $k_{k}= \beta_{0}+ {\beta_{1}.\mu}_{j}$

where $\mu_{j}$represents the average baseline FECs (in EPG) for school $j$ and $k_{k}$ is the inter-individual variability within schools.

**References**

1. Denwood MJ, Love S, Innocent GT, Matthews L, McKendrick IJ, Hillary N, et al. Quantifying the sources of variability in equine faecal egg counts: implications for improving the utility of the method. Vet Parasitol. 2012;188: 120–126.

2. Cools P, Vlaminck J, Albonico M, Ame S, Ayana M, José Antonio BP, et al. Diagnostic performance of a single and duplicate Kato-Katz, Mini-FLOTAC, FECPAKG2 and qPCR for the detection and quantification of soil-transmitted helminths in three endemic countries. PLoS Negl Trop Dis. 2019;13: e0007446.

3. Leta GT, Mekete K, Wuletaw Y, Gebretsadik A, Sime H, Mekasha S, et al. National mapping of soil-transmitted helminth and schistosome infections in Ethiopia. Parasit Vectors. 2020;13: 1–13.
