# Supplementary material for: A general framework to support cost-efficient survey design choices for the control of soil-transmitted helminths when deploying Kato-Katz thick smear
Source: PLoS Negl Trop Dis. 2023 Jun 22;17(6):e0011160. doi: 10.1371/journal.pntd.0011160 (PMC10321644; doi:10.1371/journal.pntd.0011160)
Supplement: S3 Info — (DOCX) [file pntd.0011160.s003.docx]

**A general framework to support cost-efficient survey design choices for the control of soil-transmitted helminths when deploying Kato-Katz thick smear**

**S3 Info: Estimation of total survey cost to monitor and evaluate STH when using Kato-Katz thick smear**

Adama Kazienga^1,2*^, Bruno Levecke^1^, Gemechu Tadesse Leta^3^, Sake J. de Vlas^2^, Luc E. Coffeng^2^

^1^Department of Translational Physiology, Infectiology and Public Health, Ghent University, Merelbeke, Belgium

^2^Department of Public Health, Erasmus MC, University Medical Center Rotterdam,

Rotterdam, The Netherlands

^3^Bacterial, Parasitic and Zoonotic Diseases Research Directorate, Ethiopian Public Health Institute, Addis Ababa, Ethiopia

### * Corresponding author[kazienga_adama@yahoo.fr](mailto:kazienga_adama@yahoo.fr).

We estimated the total survey cost per soil-transmitted helminth (STH) species and infection intensity level (any intensity or moderate-to-heavy intensity (MHI) infections). This total cost ($C_{total}$) comprised $(i)$ the cost of consumables to collect and process samples ($Cost_{consumables}$), and the operational costs to both $(ii)$ collect and process the samples (${Cost}_{collect/process}$) and (iii) to inform the schools about the study (${Cost}_{infoschools}$). For this, we assumed that a team (comprised of one nurse and three laboratory technicians) collects samples in the morning (8:00 – 12:00), and in the afternoon (13:00 – 17:00), all collected samples are processed [1]. Also, the number of samples that can be collected daily is limited. Therefore, we first estimated the required number of days ($n_{days})$, required to screen all recruited subjects. This number will depend on the number of samples (number of schools, number of children per school, number of samples and smears per stool sample) and the time needed to count eggs. The latter will vary by intensity of infection level (any intensity *vs.* MHI infections). We further assumed that (i) there was no additional cost for the laboratory infrastructure, (ii) the team did not take any break during processing, and (iii) the team only worked on regular working days (Monday – Friday). The equation to calculate the total survey cost for a simulated survey design, infection intensity level (any intensity or MHI infections), and STH species ($C_{total}$) was defined as follows:

$Cost_{total}=Cost_{consumables}+{Cost}_{collect/process}+Cost_{infoschools}$ **Eq. (1)**

In the following three sections, we provided technical details of the cost of consumables ($Cost_{consumables}$), sample collection and processing (${Cost}_{collect/process}$), and informing the schools (${Cost}_{infoschools}$).

**Cost of consumables to collect and process samples**

We defined the consumables costs to collect and process samples for a simulated survey design $a$x$b$ ($a$represents the number of stool samples per person, and $b$ is the number of repeated smears per stool sample) as follows:

$Cost_{consumables}=n_{school}$x$n_{children}$x$a (cost_{sample}+b$x${cost}_{aliquot,b})$ **Eq. (2)**

Here, $n_{school}$ represents the number of samples schools, $n_{children}$, the number of children per school, $cost_{sample}$is the consumable cost to collect a single stool sample, and ${cost}_{aliquot,b}$ is the cost per aliquot when processing a set of $b$aliquots from the same stool sample. **Table A in S3 Info** presents the cost per unit of consumables to collect and process samples [2].

**Table A. Overview of cost per unit of consumables to collect and process samples**

| **Variables** | **Number of aliquots per stool sample (**$\boldsymbol{b}$**)** | **Description** | **Cost (US$)** |
| --- | --- | --- | --- |
| $cost_{sample}$ |  | Cost of consumables to collect a single stool sample | 0.57 |
| ${cost}_{aliquot,b}$ | 1 | Cost of materials for a single aliquot from the same stool sample | 1.37 |
|  | 2 | Cost of materials for duplicate aliquot from the same stool sample | 0.755 |

**Operational costs of collecting and processing samples**

The operational costs of collecting and processing samples depends on the number of days $Ndays$to complete a survey, the number of mobile laboratory teams ($n_{team})$ and their daily salary ($Cost_{salary})$. It also includes the cost of transport, which also depends on the number of surveys days and is comprised of the car rental, gasoline, and drivers’ wages ($Cost_{drivers}$). We considered one mobile team comprised of one nurse and three laboratory technicians. The daily salary per mobile team member ($Cost_{salary}$) was set to be 22.5 $US\$$. In addition, the daily cost of car rental, including petrol and driver’s wages ($Cost_{drivers}$) was set to 90 $US\$$ [3].

The operational cost was estimated as follows:

${Cost}_{collect/process}= Ndays_{a\times b}$x$(n_{team}$x$Cost_{salary}+Cost_{drivers})$ **Eq. (3)**

The number of days ($Ndays_{a\times b}$) needed to screen all recruited participants for a simulated survey design $KK_{a \times b}$ was estimated as the required total time to process all samples divided by the person-time available per day to perform the work ($T_{workday})$. The total time to process all samples for a given survey is composed of the time to enter demographic data ($Time_{demo})$, to prepare $b$ aliquots samples ($Time_{prep})$, to count the eggs ($Time_{count,b}$) and to enter the results ($Time_{res})$. Note that this time also depends on the number of schools, the number of children per school, and the number of aliquots.

Therefore, we defined the number of days to carry out a simulated survey design as follows:

$\mathrm{Ndays}_{\mathrm{axb}}=\sum_{j=1}^{n_{schools}} \left\lceil\frac{n_{children} x \left( Time_{demo} + b x \left( Time_{prep}+Time_{res} \right) \right)+ \sum Time_{count,b}}{T_{workday}} \right\rceil$

+ $\left( a-1 \right)$x$\left\lceil\frac{n_{children} x b x \left( Time_{prep}+Time_{res} \right)+ \sum Time_{count,b}}{T_{workday}} \right\rceil$ **Eq. (4)**

Here, the total person-time available per day to process the samples ($T_{workday}$) depends on the number of laboratory technicians ($N_{reader}),$ and was defined as:

$T_{worday}=N_{reader}$x$4h$x$60$min x 60 sec

The required time to count eggs ($Time_{count,b}$) in $b$aliquots of $N$ stool samples depends on the goal of the survey (e.g., detecting infection (counting at least one egg) or quantifying intensity of infection (counting all eggs). Here we assumed that the objective of the survey is to quantify the intensity of infection by counting all eggs in each aliquot and that we are only considering one STH species at a time. For this, we used a function $f(c)$ that returns the time required to count $c$eggs from aliquot $i =\left( 1, 2, .., b \right)$ and individual $j=\left( 1,2, .. N \right).$ This function was derived in previous work (Coffeng LE, Vlaminck J, Cools P, et al, manuscript accepted by Plos NTD) and was defined as follows:

| $Time_{count,b}=\sum_{i=1}^{N} \sum_{j=1}^{b} f\left( \text{c}_{ij} \right)$ | **Eq. (5)** |
| --- | --- |

**Table B in S3 Info** shows the parameters used to determine the time required to process a single stool sample**.**

**Table B. Overview of parameters that determine the time required to process a single stool sample**

| **Variables** | **Description** | **Average required time (seconds)** |
| --- | --- | --- |
| $Time_{demo}$ | Time to enter demographic data | 15 |
| $Time_{prep}$ | Time to prepare a single aliquot sample | 67 |
| f(c) | Time required to count $c$ eggs (not EPG) in a single aliquot | $10^ \left( 2.3896+0.0661 x \log_{10} \left( c+1 \right)^{2} \right)$ |
| $Time_{res}$ | Time to record count data from a single aliquot sample | 9 |

**Operational costs to inform the schools**

This cost concerns the time required to inform the schools of the purpose of the study. It depends on the number of schools ($n_{schools})$, the number of mobile laboratory teams ($n_{team}$), their daily salary ($Cost_{salary})$ as well as the per diem for the car rental and drivers’ wages ($Cost_{drivers})$. This cost was defined as follows:

$Cost_{infoschools}=n_{school} x (n_{team} x Cost_{salary}+Cost_{drivers})$ **Eq. (6)**

**References**

1. Kazienga A, Coffeng LE, de Vlas SJ, Levecke B. Two-stage lot quality assurance sampling framework for monitoring and evaluation of neglected tropical diseases, allowing for imperfect diagnostics and spatial heterogeneity. PLoS Negl Trop Dis **2022**; 16:e0010353.

2. Coffeng LE, Vlaminck J, Cools P, et al. A general framework to support cost-efficient fecal egg count method and study design choices for large-scale STH deworming programs – monitoring of therapeutic drug efficacy as a case study. PLoS Negl Trop Dis **2023** doi:Forthcoming;

3. Leta GT, Mekete K, Wuletaw Y, et al. National mapping of soil-transmitted helminth and schistosome infections in Ethiopia. Parasit Vectors **2020**; 13:1–13.
