# Supplementary figures and images for: A general framework to support cost-efficient survey design choices for the control of soil-transmitted helminths when deploying Kato-Katz thick smear
Source: PLoS Negl Trop Dis. 2023 Jun 22;17(6):e0011160. doi: 10.1371/journal.pntd.0011160 (PMC10321644; doi:10.1371/journal.pntd.0011160)

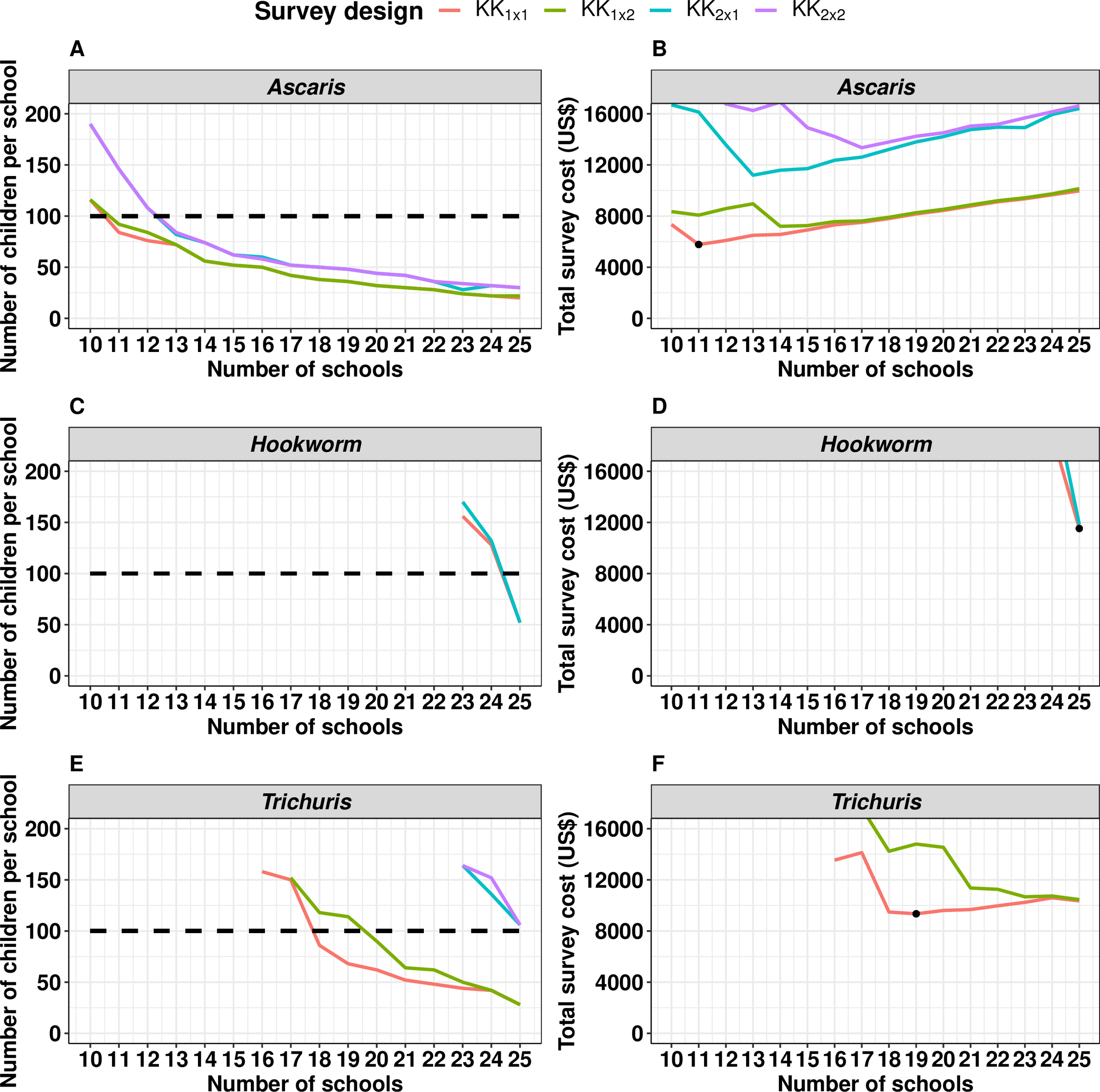

Supplement: S1 Fig — This figure illustrates the required number of children per school (nchildren; Panels A, C and E) and the corresponding total survey cost (Ctot; Panels B, D and F) as a function of the number of sampled schools (nschools) for different survey designs and soil-transmitted helminths (Ascaris: Panels A and B; hookworm: Panels C and D; Trichuris: Panels E and F). The survey designs (KKa×b) varied in the number of stool samples per child (= a) and the number of Kato-Katz thick smears per sample (= b). The black bullet point in Panels B, D and F indicates the most cost-efficient survey design. In other words, the number of schools that minimizes the costs while ensuring reliable decision-making. Note that we assumed that the maximum number of children per school could not exceed 100. (TIF) [file pntd.0011160.s004.tif]
