# Supplementary material for: A general framework to support cost-efficient survey design choices for the control of soil-transmitted helminths when deploying Kato-Katz thick smear
Source: PLoS Negl Trop Dis. 2023 Jun 22;17(6):e0011160. doi: 10.1371/journal.pntd.0011160 (PMC10321644; doi:10.1371/journal.pntd.0011160)
Supplement: S1 Table — This table presents the sample size (nschools x nchildren), the decision cut-off c and the associated total survey costs (Ctot) for different scenarios of parameterizing the aggregation of infections within children (kk), the clinical specificity of Kato-Katz thick smear and the maximum allowed risk of incorrect decision-making for the different soil-transmitted helminths species separately. As a reference we assumed that kk varies as a function of school mean eggs per gram of stool (EPG) and that Kato-Katz thick smears has a perfect specificity. We allowed for a risk of undertreatment (Eundertreat) equal to 0.05 and risk of overtreatment (Eovertreat) equal to 0.25. In addition, the fixed aggregation parameter (Fixed kk), indicating the variation between individuals within schools was 0.326 for Ascaris, 0.257 for hookworm, and 0.532 for Trichuris [6]. A lower value of kk (Lower kk) means higher inter-individual variation in EPG values, assumed to be 2/3 of kk in the main analysis. (DOCX) [file pntd.0011160.s005.docx]

**S1 Table. The impact of the model assumptions on both the required sample size and the total survey costs to reliably declare elimination of soil-transmitted helminths as a public health problem.**

| **Parameters** | | ***Ascaris*** | | |  | **Hookworm** | | |  | ***Trichuris*** | | |
| --- | --- | --- | --- | --- | --- | --- | --- | --- | --- | --- | --- | --- |
|  |  | Sample size | $c$ | $C_{tot}$ ($US\$)$ |  | Sample size | $c$ | $C_{tot}$ ($US\$)$ |  | Sample size | $c$ | $C_{tot}$ ($US\$)$ |
| ***Aggregation of infections within children (***$\boldsymbol{k}_{\boldsymbol{k}}$***)*** | | | | | | | | | | | | |
|  | Main analysis | 11 x 84 | 13 | 5,775 |  | 25 x 52 | 17 | 11,522 |  | 19 x 68 | 16 | 9,346 |
|  | Varying $k_{k}$ as a linear function of school mean EPG | | | | | | | | | | | |
|  | Fixed $k_{k}$ | 10 x 64 | 9 | 4,841 |  | 13 x 60 | 11 | 6,193 |  | 11 x 62 | 10 | 5,283 |
|  | Lower $k_{k}$ | 12 x 70 | 12 | 5,950 |  | 27 x 32 | 27 | 13,492 |  | 20 x 78 | 21 | 10,226 |
|  |  |  |  |  |  |  |  |  |  |  |  |  |
| ***Risks of incorrect decision-making*** | | | | | | | | | | | | |
|  | $E_{undertreat}=0.025; E_{overtreat}=0.25$ | 15 x 88 | 18 | 8,048 |  | 33 x 68 | 29 | 16,233 |  | 26 x 62 | 22 | 12,487 |
|  | $E_{undertreat}=0.025; E_{overtreat}=0.10$ | 22 x 86 | 30 | 11,666 |  | 49 x 96 | 71 | 27,121 |  | 36 x 90 | 51 | 19,245 |
