# Supplementary material for: A general framework to support cost-efficient survey design choices for the control of soil-transmitted helminths when deploying Kato-Katz thick smear
Source: PLoS Negl Trop Dis. 2023 Jun 22;17(6):e0011160. doi: 10.1371/journal.pntd.0011160 (PMC10321644; doi:10.1371/journal.pntd.0011160)
Supplement: S2 Table — This table summarizes the required sample size (nschools x nchildren) and the corresponding cut-off c (maximum number of positive individuals) across different scenarios of mono and mixed soil-transmitted helminths (STH) infections. This table only applies to surveys that perform a duplicate Kato-Katz thick smear on a single stool sample. (DOCX) [file pntd.0011160.s006.docx]

**S2 Table. The recommended sample size and corresponding soil-transmitted helminth specific decision cut-off for reliably stopping preventive chemotherapy based on predominant helminth species within an implementation unit.**

|  | | $\boldsymbol{n}_{\boldsymbol{schools}}\boldsymbol{x}\boldsymbol{n}_{\boldsymbol{children}}$ | | **STH-specific decision cut-off** $\boldsymbol{c}$ | | |
| --- | --- | --- | --- | --- | --- | --- |
|  |  |  |  | *Ascaris* | Hookworm | *Trichuris* |
| ***Mono-infections*** | | | | | | |
|  | *Ascaris* | 5 | 64 | 7 | _ | _ |
|  | Hookworm | 6 | 66 | _ | 9 | _ |
|  | *Trichuris* | 4 | 70 | _ | _ | 7 |
| ***Infections with 2 STH*** | | | | | | |
|  | *Ascaris* & hookworm | 6 | 66 | 8 | 9 | _ |
|  | *Ascaris* & *Trichuris* | 5 | 64 | 7 | _ | 8 |
|  | Hookworm & *Trichuris* | 6 | 66 |  | 9 | 9 |
| ***Infections with all STH*** | | | | | | |
|  |  | 6 | 66 | 8 | 9 | 9 |
